# Case Report: Vascular access in paediatric haemodialysis patients—creating and maintaining the patency of an arteriovenous fistula

**DOI:** 10.3389/fsurg.2024.1181802

**Published:** 2024-03-19

**Authors:** Ramona Stroescu, Serban Comsa, Flavia Chisavu, Mihai Gafencu

**Affiliations:** ^1^ “Victor Babes” University of Medicine and Pharmacy, Timișoara, Romania; ^2^ 4th Pediatric Clinic, “Louis Țurcanu” Children's Clinical Sand Emergency Hospital, Timișoara, Romania; ^3^ Department of Microscopic Morphology/Histology, Angiogenesis Research Center, “Victor Babes” University of Medicine and Pharmacy, Timișoara, Romania; ^4^ Faculty of Medicine “Victor Babes”, Centre for Molecular Research in Nephrology and Vascular Disease, Timișoara, Romania

**Keywords:** arteriovenous fistula, hemodialysis, thrombosis, children, surgeon

## Abstract

**Introduction:**

Paediatric vascular access is a demanding field. The need for a multidisciplinary team is mandatory in children with end-stage kidney disease (ESKD). Central venous catheters (CVCs) remain the preferred option worldwide. Recent emerging data demonstrated the benefits of using arteriovenous fistulas (AVFs) in the paediatric population for long-term vascular access. The small vessel size in children represents a surgical challenge for vascular access.

**Case presentation:**

We report three cases from our haemodialysis department and the difficulty in maintaining permanent vascular access. The first case is an adolescent girl who required a change in vascular approach after multiple central venous catheter (CVC) infections and catheter thrombosis secondary to thrombophilia. Three AVFs were performed but failure occurred early. The patient was also diagnosed with a complex vascular thrombosis with total occlusion of the inferior vena cava and completed distal thrombosis of the superior vena cava. A permanent CVC was placed in the right jugular vein with the tip in the azygos vein. The second case is of an adolescent boy with systemic vasculitis with multiple CVC infections secondary to immunosuppression. The first thrombosis of two right AVFs occurred early with the development of a pseudo-aneurysm that required surgical intervention. The left brachial-cephalic fistula required surgery for closing the collaterals, repositioning and superficialisation. The third case is an adolescent boy with one surgical stage brachial-basilic left AVF and difficulties in venous puncturing.

**Conclusion:**

Vascular access in paediatric haemodialysis remains a demanding field. There is a need for a multidisciplinary team, consisting of a vascular surgeon and an interventional radiologist specialising in children.

## Introduction

Paediatric haemodialysis access is a demanding field. Unlike adults, children require vascular access adjusted to their growing rhythm. This underlines the importance of a multidisciplinary team with expertise in all aspects of care for a child with renal failure. The guidelines of the National Kidney Foundation's Kidney Disease Outcomes Quality Initiative (NFK-KDOQI) recommend a permanent access placement in dialysis patients aged 0–19 years old who weigh over 20 kg and are unlikely to receive a transplant in less than 1 year (1).

The most important assay in children with chronic kidney disease (CKD) is vein preservation. The three main paediatric long-term vascular access options are central venous catheter (CVC), arteriovenous fistula (AVF), and arteriovenous graft (AVG). Despite the clear advantages of using AVF, CVCs remain the first option according to the International Paediatric Haemodialysis Network (IPHN) registry (2).

The last decade has seen significant advancement in paediatric renal replacement therapy focused on improving vascular access in children of all ages to achieve effective haemodialysis, promote normal growth, and enhance overall quality of life. Vascular access remains a surgical challenge because of the vessel size and the unique physiology of patients. Although kidney transplantation remains the gold standard, patent vascular access is mandatory for adequate dialysis.

We present three patients from our haemodialysis department with impaired vascular access and difficulty in maintaining primary and secondary patency of the AVF due to individual risk factors. The major setback was represented by the lack of specialised paediatric vascular surgeons and interventional radiologists.

These case reports involving human participants were reviewed and approved by the ethics committee of the Emergency Hospital for Children Louis Turcanu. Written informed consent was obtained from all legal guardians of the the minors for the publication of any potentially identifiable images or data included in this article.

## Case 1

We report the case of a 17-year-old girl with stage V CKD, secondary to congenital anomalies of the kidney and urinary tract (CAKUT) with preserved diuresis (2,500 ml/day), which is currently receiving thrice weekly haemodialysis. She weighed 1,600 g at birth, which was small for her gestational age of 36 weeks, and had impaired renal function from the first day of life secondary to urogenital sinus, neurogenic bladder, and reflux nephropathy with bilateral ureterohydronephrosis that progressed to end-stage kidney disease (ESKD). She was initiated on continuous ambulatory peritoneal dialysis at the age of 4 years, and she was switched to haemodialysis due to recurrent fungal peritonitis at the age of 10 years. Due to multiple CVC infections and catheter thrombosis, there was an imperative need for a different haemodialysis access. In 2020, at the age of 15 years, she underwent a left radio-cephalic AVF performed by a vascular surgeon specialised in adults but with no success as fistula thrombosis occurred within 7 days. A genetic profile for thrombophilia was performed. Several factors were identified with a high risk of associated thrombophilia: Factor V G1691A (Leiden) heterozygous mutation, MTHFR C677T heterozygous mutation, Factor XIII V34l heterozygous mutation, and PAI-5G/5G homozygous. The anticoagulation of the patient was performed with enoxaparin in a therapeutic dose.

**Figure 1:**
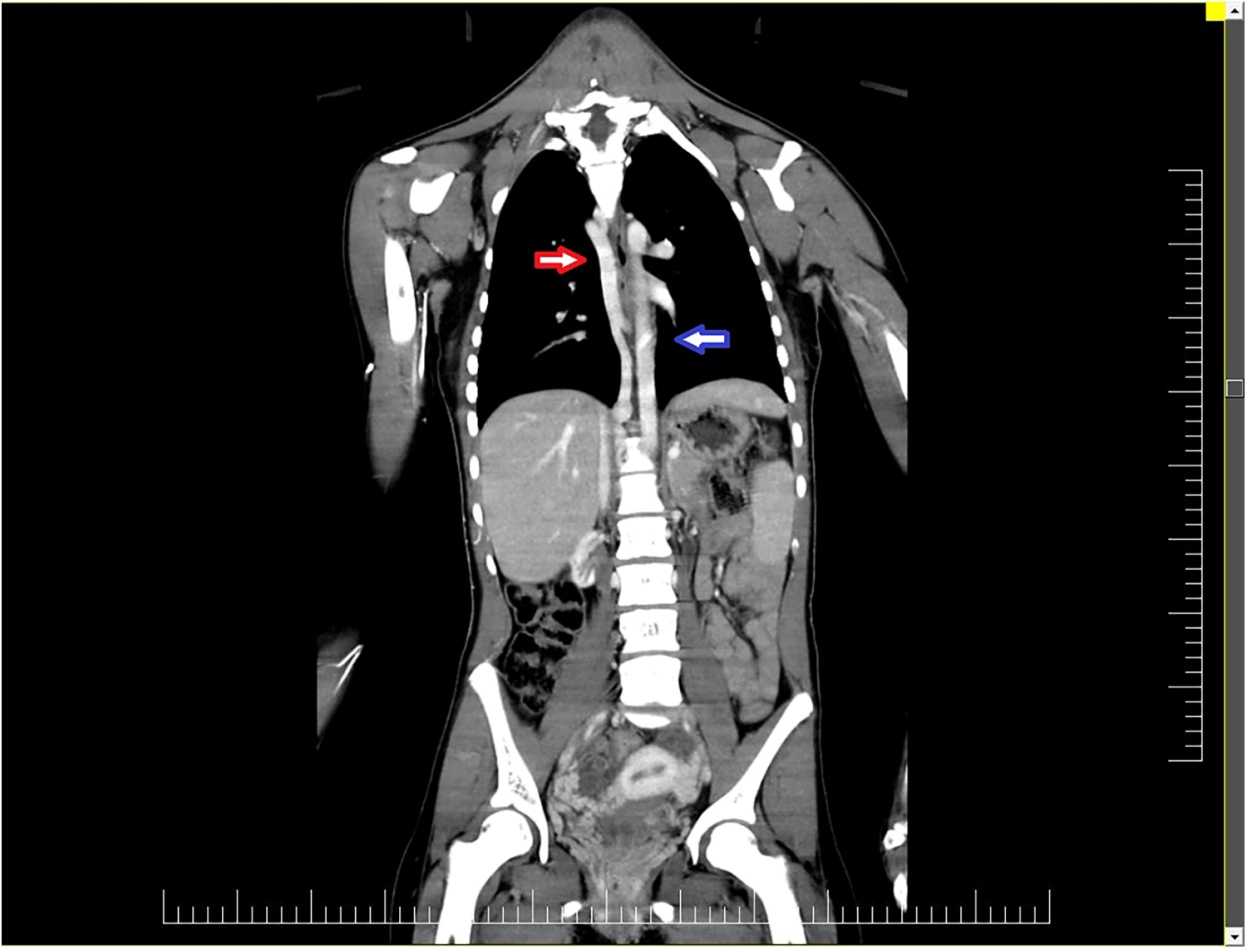
CT angiogram revealed numerous thrombosis sites. The superior vena cava displayed an area of complete distal thrombosis between the right atrium shedding and the azygos vein cross opening in the superior vena cava, on a length of 2.8 cm, with dilated azygos (red arrow) and hemiazygos veins (blue arrow).

CT angiogram revealed multiple thrombosis sites. The superior vena cava displayed an area of complete distal thrombosis proximal to the right limb shedding on a length of 2.8 cm with dilated azygos and hemiazygos veins and the azygos vein cross opening in the superior vena cava above the thrombosis (Figure 1).

**Figure 2:**
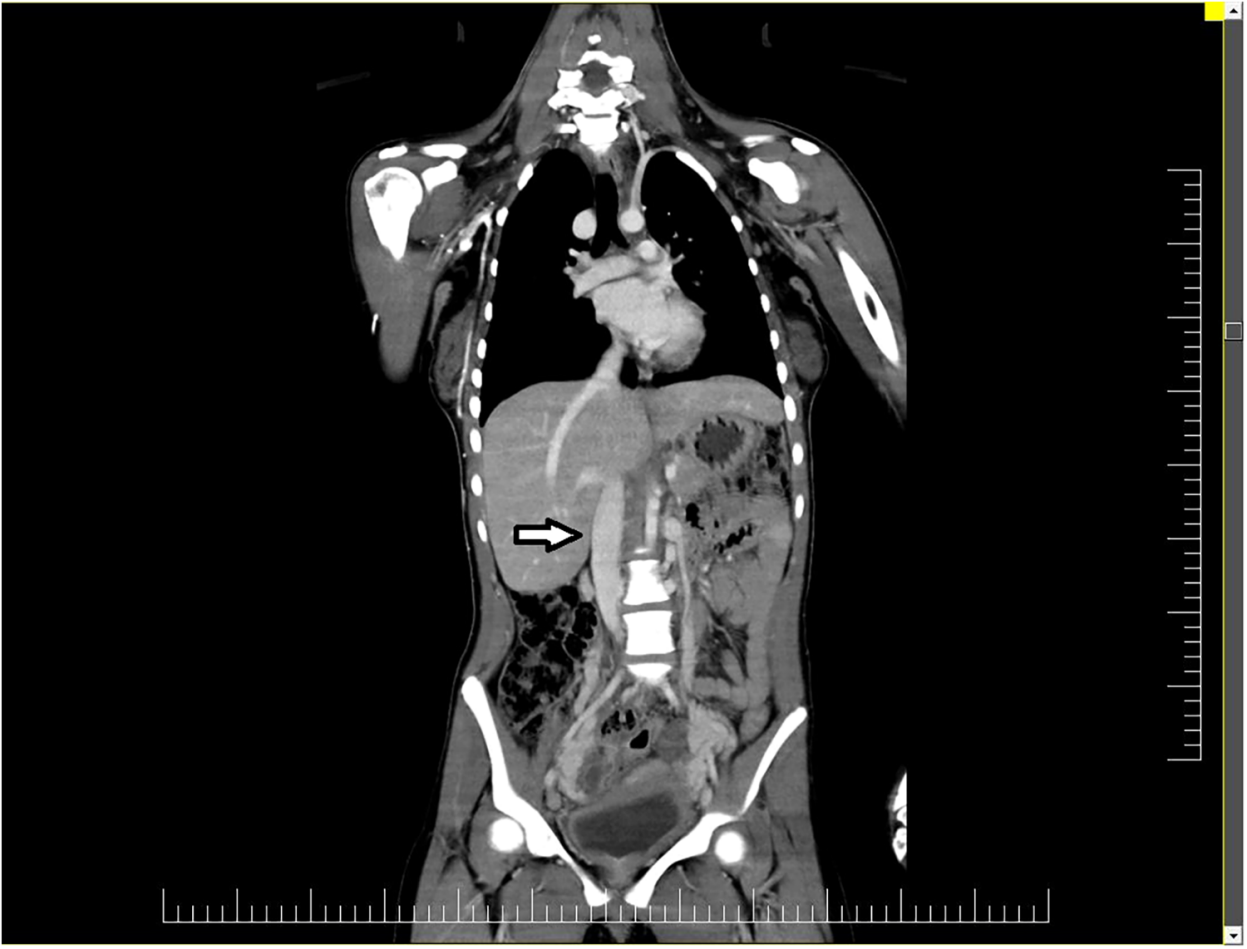
The inferior vena cava (arrow) showed significant decalibration near the L4 vertebral body with no lumen detected.

The inferior vena cava showed significant decalibration near the L4 vertebral body with no lumen detected. Numerous serpiginous venous trajectories (venous collaterals) drain the blood from the lower extremity into the paravertebral veins and through them in the inferior vena cava through the subhepatic and retrohepatic segment (Figure 2).

After careful consideration, in 2021, another AVF was done on the non-dominant hand of the patient between the brachial artery and the basilica vein with primary patency obtained under strict anticoagulation. The fistula was punctured using a 17-gauge single needle because only 2 cm was prone to use. After 3 months, AVF thrombosis occurred requiring a temporary CVC in the right jugular vein. In the given circumstances, a third AVF was done on the dominant hand of the patient between the brachial artery and the cephalic vein because the catheter was the last patent vascular access. Although the anticoagulation therapy was in the therapeutic range, the presence of a CVC on the ipsilateral side associated with major superior vena cava thrombosis ultimately led to AVF failure in the first weeks.

Our team faced difficulty in obtaining another catheter 4 months after CVC insertion when signs of catheter thrombosis were present. We addressed the patient in an outer clinic to a mixed team consisting of a vascular surgeon and an interventional radiologist for a permanent catheter placement under the fluoroscope. The team decided to insert a permanent catheter in the right jugular vein with the tip in the azygos vein with the description of the procedure below.

### Surgical procedure

The femoral artery was punctured, and a 0.035″ guide wire and consecutively a vertebral catheter were advanced towards the right internal carotid artery. An iodinated contrast medium was injected through the vertebral catheter to obtain the venogram of the superior vena cava territory. Occlusion of the terminal superior vena cava with compensatory dilatation of the azygos vein was documented. The azygos vein had an inverted flux, facilitating the blood drainage from the superior vena cava territory to the inferior vena cava. Another 0.035″ guide wire was inserted in the temporary right internal jugular vein catheter and advanced through the vena cava towards the azygos vein followed by catheter removal. A 14 French permanent catheter was tunnelled in the right pre-pectoral zone and was advanced through the sheath over the wire in the azygos vein. Its patency was tested just before locking it with heparin. Postoperative anticoagulation was continued with low-molecular-weight heparin (LMWH) in therapeutic ranges, with the first dose administered 8 h after the surgical procedure.

The permanent azygos catheter is still in use today. The patient had a low chance of kidney transplantation.

## Case 2

A 15-year-old boy addressed the nephrology department in June 2020 with ESKD, secondary to ANCA vasculitis. In 2017, he was diagnosed with myeloperoxidase anti-neutrophil cytoplasmic antibody (ANCA-MPO) vasculitis. He was initially treated in the haematology clinic for severe haemolysis episodes with haematuria and nephrotic range proteinuria. The patient repeatedly refused kidney biopsy and was non-compliant with the immunosuppressive treatment due to ethnical and religious reasons. Keeping in mind the acute need for renal replacement therapy, a temporary CVC was placed, and he continued with thrice weekly haemodialysis and immunosuppression treatment according to vasculitis guidelines after three sessions of plasmapheresis.

In August 2020, after achieving remission, a right radio-cephalic AVF fistula was performed, in the same operatory time with the insertion of a permanent catheter. Due to vessels being frailty, the primary patency was not achieved. In December 2020, vasculitis reactivation occurred due to non-compliance to the remission-maintaining therapy, requiring the resumption of the induction scheme.

**Figure 3:**
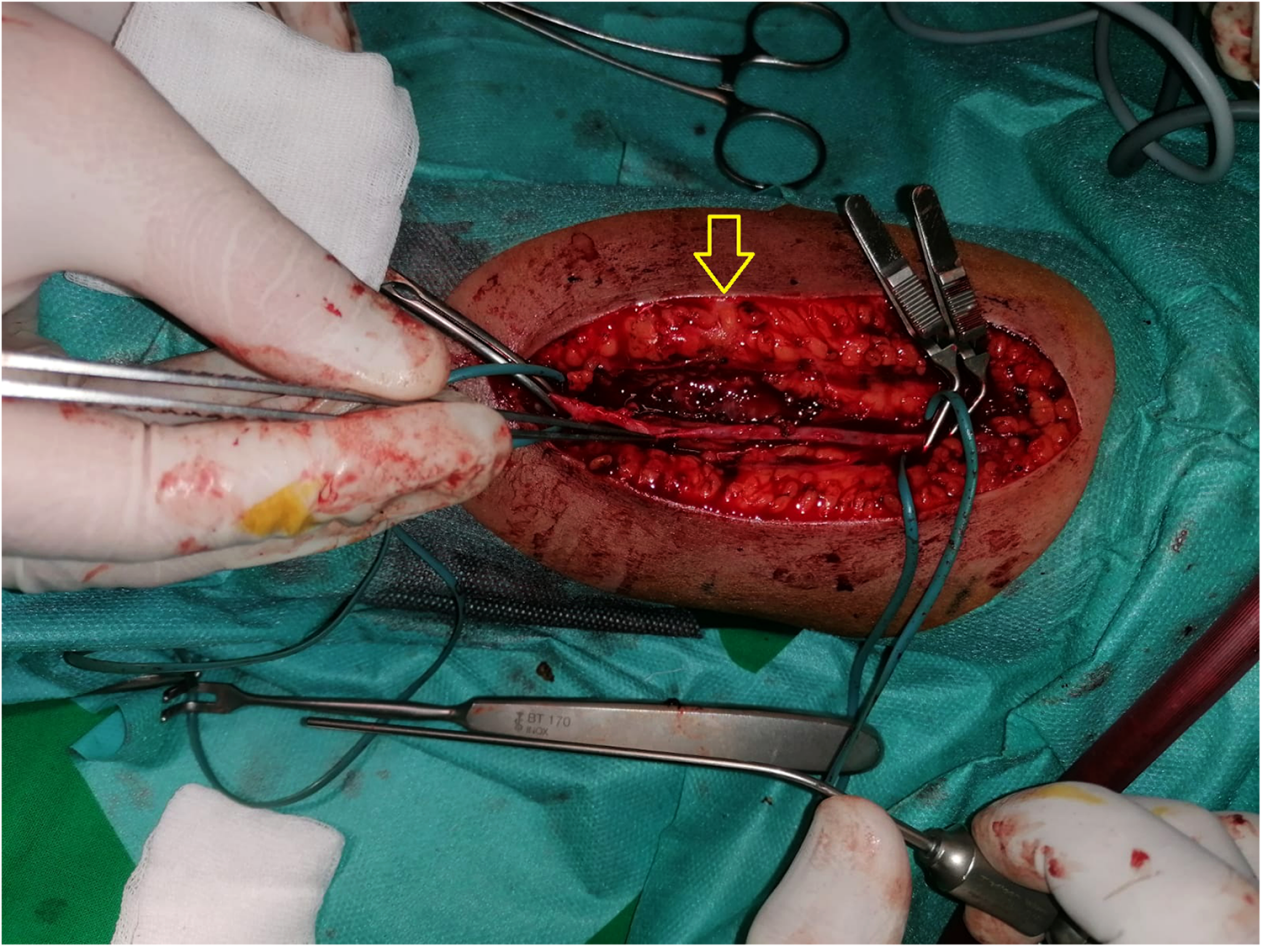
Arteriovenous fistula (AVF) with pseudo-aneurysm (arrow) developed on the brachial artery followed by complete thrombosis.

In April 2021, after a septic shock secondary to catheter infection, it was considered appropriate to create a new fistula. An AVF fistula was done on the same side as the previous one, between the brachial artery and the cephalic vein, with successful maturation. After the first fistula puncture, a pseudo-aneurysm developed followed by complete thrombosis (Figure 3).

The evolution was marked by multiple catheter infections with the need to change the vascular access. In October 2021, a third AVF fistula was created in the non-dominant hand of the patient between the brachial artery and the cephalic vein with primary patency achieved. The evolution was marked by a new pseudo-aneurysm development, without haemodynamic impact but with a turbulent flow and increasing size. This pseudo-aneurysm appeared due to a 4 mm linear lesion of the posterior wall of the post-fistular vein. Possible reasons could be an accidental puncture of the posterior wall in an inside–outside manner, a poor quality of the venous wall, or a mixed mechanism. Six weeks later, in December 2021, after careful evaluation, the patient underwent an operation to close the brachial-cephalic left fistula collaterals with repositioning and superficialisation as mentioned below. Another surgical intervention was mandated in March 2022 on the previously thrombosed right AVF due to the increasing size of the pseudo-aneurysm that presented significant haemodynamic impact.

### Surgical procedure

**Figure 4:**
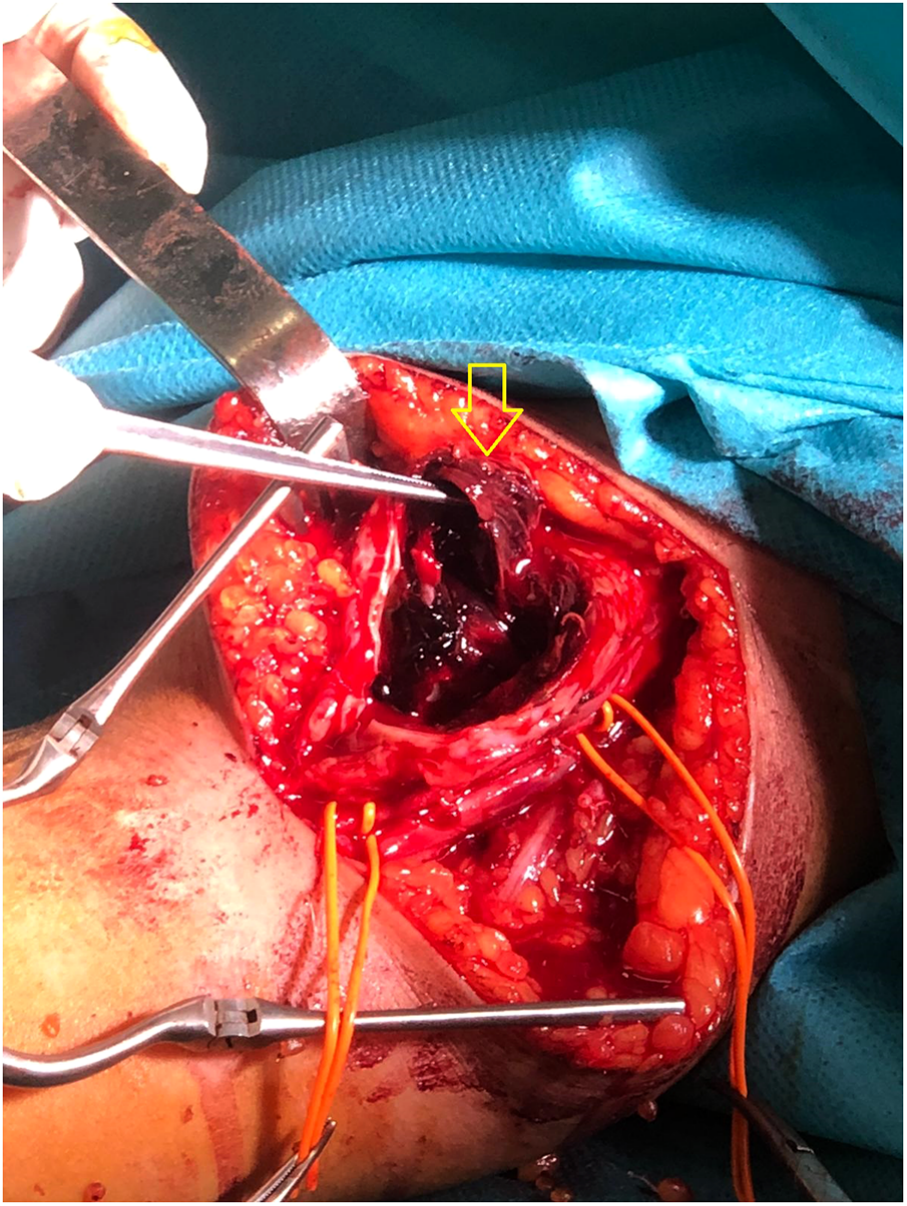
During surgery, a 4 mm linear lesion was identified on the posterior wall of the post fistula vein in the middle third of the arm (arrow), feeding a 7/3 cm bisacular intramuscular pseudo-aneurysm located posterior to the vein (not visible in the image).

During surgery, a 4 mm linear lesion was identified on the posterior wall of the post-fistular vein in the middle third of the arm, feeding a 7/3 cm bisacular intramuscular pseudo-aneurysm located posterior of the vein (Figure 4).

As the diameter of the vein was not more than 4 mm at the level of the lesion, a direct suture of the lesion was avoided. Instead, a segment of the vein that included the lesion was excised, the vein was dissected proximally and distally, and a terminal–terminal anastomosis of the venous endings with simple interrupted sutures was performed. A lateral subcutaneous pocket was consecutively created, allowing the vein transposition and superficialisation.

In 2022, the patient had yet suffered another complication—he developed a traumatic compressive haematoma on the right upper limb, which overlapped the pre-existent vascular condition (the increasing pseudo-aneurysm), that required urgent surgical intervention, requiring a temporary haemodialysis catheter. Due to his underlying disease, all the described procedures led to a longer healing time with keloid scars.

Today, the patient is continuing haemodialysis three times a week on the ladder fistula with two 16-gauge needles. Fistula functionality remains a matter of time due to the aneurysmal vein development with the kinking area and theft syndrome from the brachial artery.

## Case 3

A 16-year-old boy addressed our clinic in 2020 with ESKD secondary to a single congenital hypoplastic left kidney. After placing a Tenckhoff catheter, continuous ambulatory peritoneal dialysis was initiated from April 2020 to April 2022 with only one episode of peritonitis due to *Staphylococcus aureus* infection. In January 2022, a brachial-basilic AVF in the non-dominant arm was created with primary and secondary patency obtained after 3 months. The fistula initially matured 5 cm above the anastomosis with a distal vein going deep under the muscle (10 mm) with a posterior orientation.

In addition to the other cases, haemodialysis was initially performed using a 17 gauge single needle, as the anatomical position could not be cannulated using two fistulas for appropriate puncturing. However, repeated punctures in a ladder manner led to an artificial superficialisation. Two months later, a safe distance between the “arterial” and “venous” sites of 6 cm was achieved. It is worth mentioning that although the AVF was functioning, only the repeated ladder punctures secured the venous tract more superficially allowing us to obtain adequate hemodialysis.

The evolution was marked by malignant hypertension after 4 months of hemodialysis. Angio-CT revealed two kidneys with filiform renal arteries in both kidneys, one sclero-atrophic, establishing the diagnosis of renal fibromuscular dysplasia. The patient underwent kidney transplantation from a deceased donor in December 2022. The AVF is still patent.

## Discussions

Vascular access in children is challenging due to the size of the blood vessels, underlying conditions, and the need to maintain long-term vascular access without compromising their vascular capital. While AVF and AVG are associated with fewer infectious complications and tend to function longer than CVCs, the development of neointimal hyperplasia leads in time to stenosis and thrombosis, thus mandating for a closer surveillance of the vascular access (3, 4).

Despite the increasing number of children receiving hemodialysis, the AVF use is underscored by the accessibility of CVCs. More often, these patients start haemodialysis abruptly due to the rapid progression to ESKD and even more in acute settings. Despite global efforts and initiatives to increase the utilisation of AFVs [e.g., The International Pediatric Fistula First Initiative (5)], CVCs remain the preferred long-term vascular access in HD paediatric patients. A radio-cephalic AVF in the non-dominant limb is commonly preferred over a brachial-cephalic AVF to preserve the vascular bed further (6). Yet, sometimes this is difficult to achieve in a child with multiple comorbidities and challenging vascular anatomy, as we encountered.

Complex vascular thrombosis reduces the vascular access options. The risk of losing vascular access increases exponentially if thrombophilia is associated with patients receiving maintenance hemodialysis. Vascular mapping and a thrombophilia genetic profile are sometimes required before placing a CVC or creating an AVF as described in the first case. Multiple CVC-related thrombosis led to haemodynamic changes in the vascular bed that decreased the chance of AVF patency. In addition, the insertion of the permanent haemodialysis catheter in the azygos vein represented a rescue intervention.

Although there are no standard anticoagulation strategies available for paediatric patients on maintenance HD at risk for thrombosis, the decision of thromboprophylaxis remains to be individualised (7).

Repeated arteriovenous fistulas (AVFs) were performed to preserve the vascular capital and to reduce CVC-related infections as described in the second case. Underlying vascular inflammation leads to vessel fragility and an increased risk of developing pseudo-aneurysms secondary to AVF puncturing.

While brachial-basilic AVF requires a two-stage approach, a ladder puncturing, as described in the third case, can help in AVF maturation, thus gaining time for a second surgical approach when needed.

The strengths of these case reports are the variety of the underlying conditions that led to vascular access difficulties. Anticoagulation does not prevent AVF or CVC thrombosis, especially in the presence of thrombophilia. To our knowledge, there are no reports regarding AVFs in paediatric patients with vasculitis on maintenance HD. Moreover, there are no reports about permanent CVCs in the azygos vein in children. Even though the brachial-basilic fistula is performed in two steps, in our case, ladder puncturing was the elegant method to increase the puncturing area. One of the drawbacks was the lack of specialised vascular surgeons and the necessity to collaborate with outer surgeons and interventional radiologists. This is a major problem worldwide, in both adults and paediatric populations, that needs to be addressed.

## Conclusions

Vascular access in paediatric haemodialysis remains a demanding field. It requires proper planning to ensure that the best permanent access is placed. CVCs are the most commonly used, but AVFs are preferred due to decreased risk of infections and their long-term use. There is a need for a multidisciplinary team—consisting of a vascular surgeon and an interventional radiologist specialising in children.

## Data Availability

The original contributions presented in the study are included in the article/Supplementary Material; further inquiries can be directed to the corresponding author.
